# Cholangioscopic rescue: A guidewire-preserving technique for retrieving an impacted plastic stent during endoscopic ultrasound-guided pancreatic duct drainage

**DOI:** 10.1055/a-2938-1232

**Published:** 2026-08-25

**Authors:** Shan-Shan Hu, Wei-Hui Liu

**Affiliations:** 1 Department of Gastroenterology and HepatologySichuan Provincial People's Hospital, School of Medicine, University of Electronic Science and Technology of ChinaChengduSichuanChina

Endoscopic ultrasound-guided pancreatic duct drainage (EUS-PD) is an established salvage therapy for pancreatic duct obstruction after failed endoscopic retrograde cholangiopancreatography (ERCP). 1​ 2​ ​3​ However, stent maldeployment during EUS-PD remains a challenging complication, particularly when a plastic stent becomes impacted in the transmural tract with the guidewire still in place. 4​ ​5​ We describe a novel cholangioscope-assisted, guidewire-preserving technique to retrieve an impacted plastic stent during EUS-PD.

**Fig. 1:**
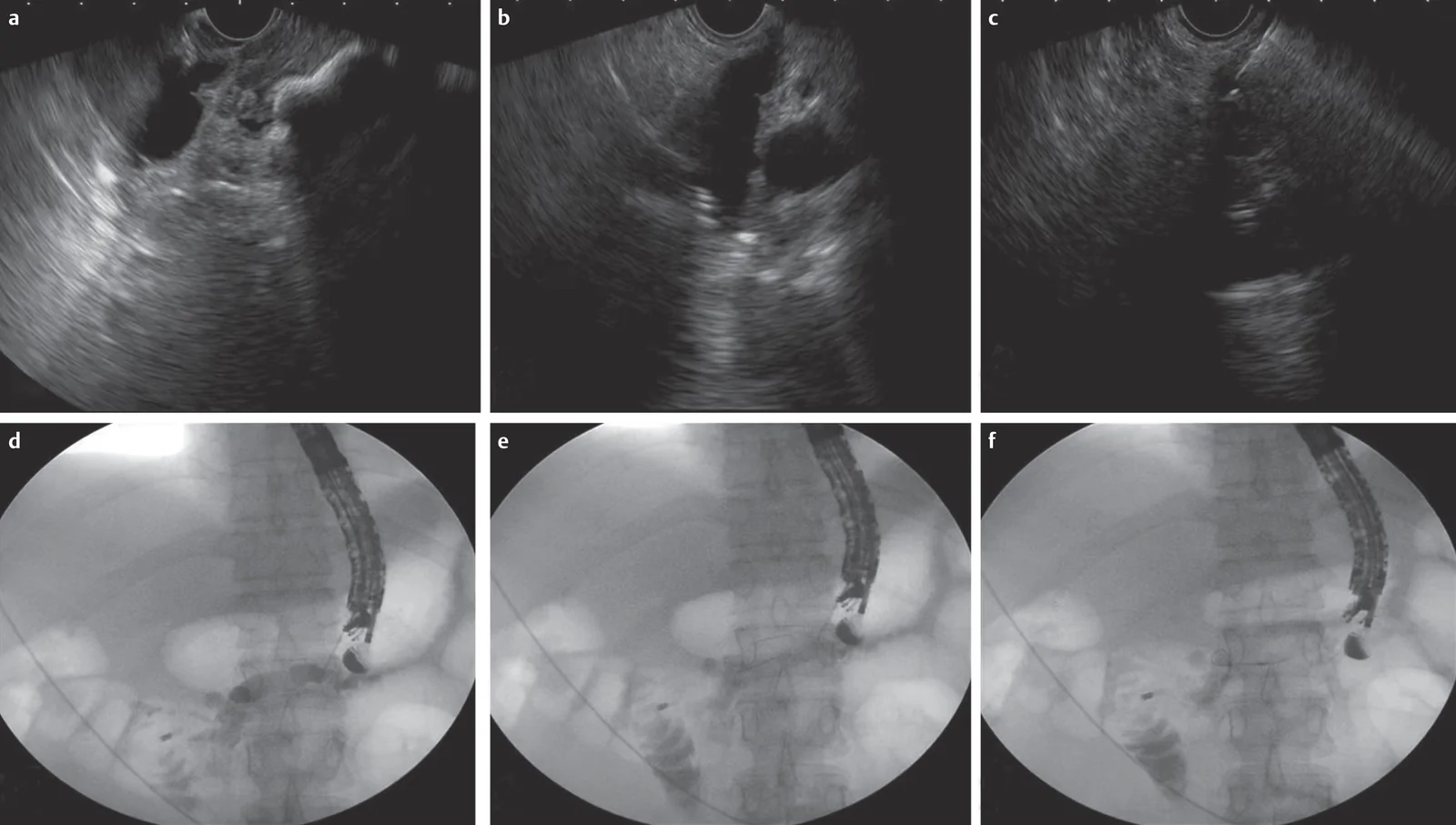
EUS-PD procedural steps. ( **a** ) EUS imaging of the pancreatic head showing multiple impacted stones in the main pancreatic duct. ( **b** ) EUS imaging of the pancreatic body and tail demonstrating marked upstream ductal dilation. ( **c** ) EUS-guided transgastric puncture of the dilated main pancreatic duct using a 19-gauge needle. ( **d** ) Contrast injection confirming pancreatic duct opacification. ( **e** ) Placement of a 0.025-inch guidewire into the pancreatic duct. ( **f** ) Fistula creation using a cystotome over the guidewire.

**Fig. 2:**
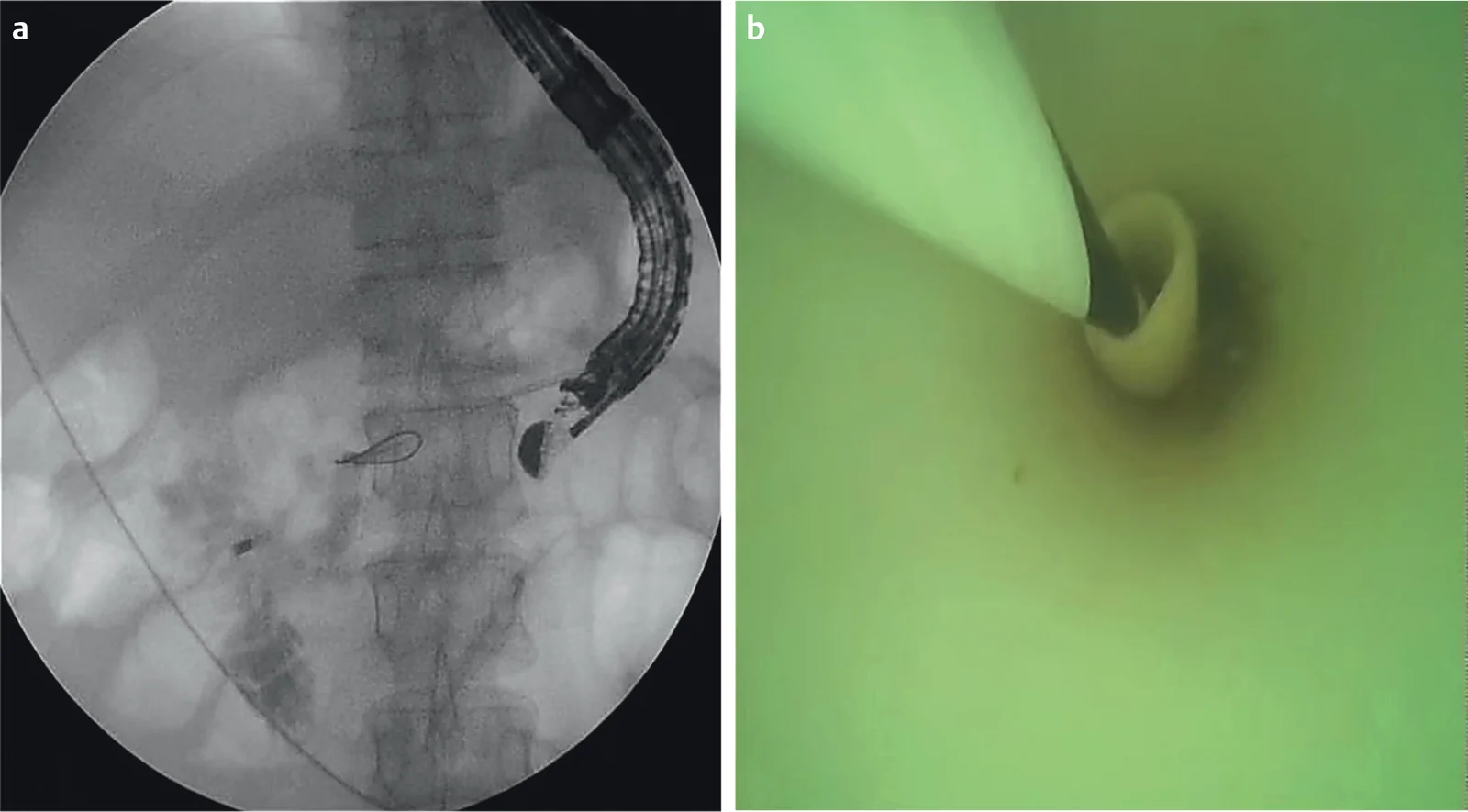
Stent impaction and cholangioscope identification. ( **a** ) The 7Fr plastic stent with a blunt tip impacted at the fistula opening; the stent body is folded in the gastro-pancreatic space, and the proximal end is lodged in the echoendoscope instrument channel. ( **b** ) The digital single-operator cholangioscope advanced through the instrument channel alongside the indwelling guidewire, providing the direct visualization of the impacted stent tip.

**Fig. 3:**
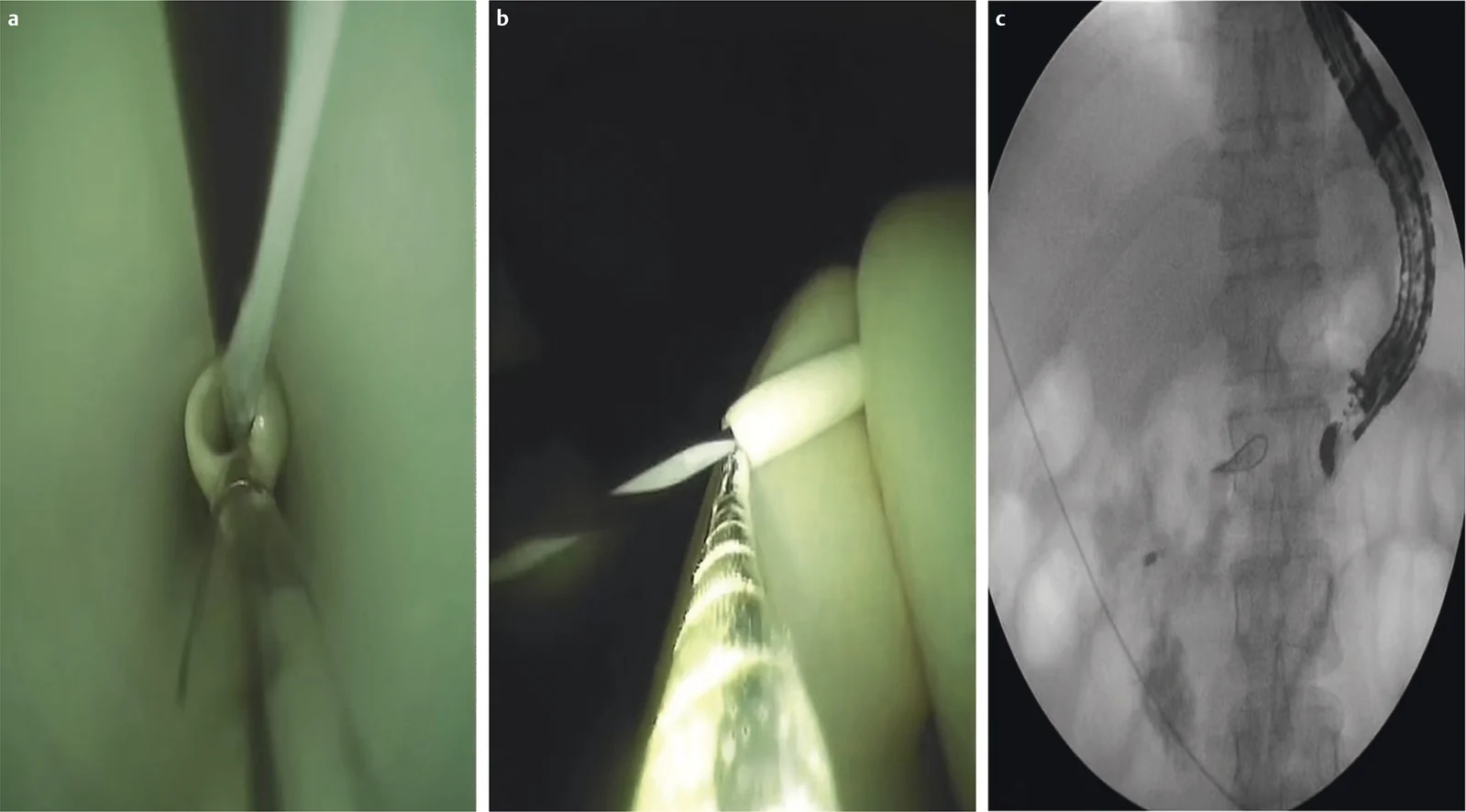
Cholangioscopic retrieval and guidewire preservation. ( **a** ) Cholangioscopic biopsy forceps grasping the stent tip under direct visualization. ( **b** ) The impacted stent being withdrawn through the instrument channel while the guidewire remains in the pancreatic duct. ( **c** ) After stent removal, the guidewire is confirmed to be in the stable position within the pancreatic duct.

**Fig. 4:**
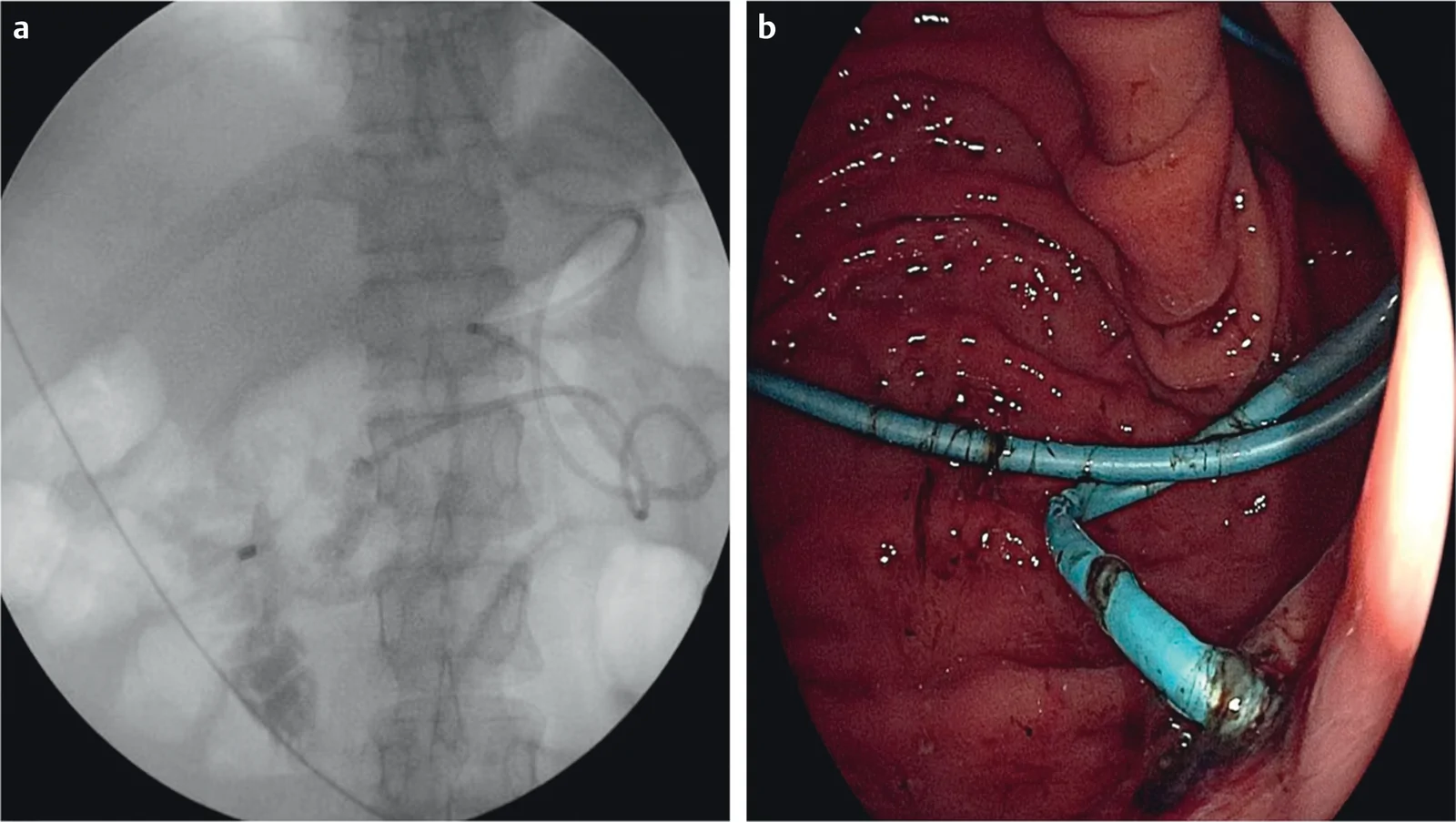
Final drainage and post-procedural confirmation. ( **a** ) A nasopancreatic drain placed over the preserved guidewire, with the fluoroscopic confirmation of the adequate position and flow. ( **b** ) An endoscopic view showing the nasopancreatic drain exiting through the gastric puncture site without active bleeding.

A 38-year-old woman with main pancreatic duct stones and ductal dilation underwent EUS-PD after failed ERCP ( Fig. 1 and Video 1 ). Following transgastric puncture and tract dilation with a cystotome, a 7Fr plastic stent was deployed but became impacted at the fistula opening, with its proximal end lodged in the echoendoscope instrument channel and its body folded in the gastro-pancreatic space ( Fig. 2a ). A digital single-operator cholangioscope was advanced through the instrument channel alongside the indwelling guidewire ( Fig. 2b ). Under direct cholangioscopic visualization within the instrument channel, the stent tip was grasped with the cholangioscopic biopsy forceps and gently withdrawn, successfully preserving the guidewire in the pancreatic duct ( Fig. 3 ). A nasopancreatic drain was then placed over the retained guidewire ( Fig. 4 ). The procedure was uncomplicated. Serum amylase and lipase remained normal at 24 hours, with no post-procedural pancreatitis, bleeding, or fistula. The nasopancreatic drain provided equivalent pancreatic drainage to a transmural stent and was maintained for 7 days to ensure adequate drainage and tract maturation, after which it was cut to form an internal drainage tract. Computed tomography at 1 week showed resolved ductal dilation without peripancreatic fluid. The patient was discharged on day 8 and remained asymptomatic at 3-month follow-up, with normalized enzyme levels and no evidence of recurrent obstruction.

**Video 1** Cholangioscopic rescue: A guidewire-preserving technique for retrieving an impacted plastic stent during endoscopic ultrasound-guided pancreatic duct drainage.


The present case highlights three critical technical considerations. First, guidewire preservation is paramount. Loss of the guidewire would result in immediate fistula collapse, necessitating repeat EUS-guided puncture with attendant risks of bleeding, perforation, and pancreatic leakage. By maintaining the guidewire in the pancreatic duct throughout the rescue maneuver, we preserved the transmural tract and enabled the seamless completion of the drainage procedure. Second, direct cholangioscopic visualization within the instrument channel allowed the precise identification of the stent tip and its relationship to the guidewire, avoiding blind traction that could cause stent fracture or ductal injury. The cholangioscope, with its 3mm outer diameter, is compatible with standard therapeutic echoendoscopes (3.8-mm instrument channel), making this technique broadly applicable without requiring specialized equipment. Third, the use of the cholangioscope's dedicated biopsy forceps through its own working channel enabled controlled grasping and axial traction, which is not feasible with conventional accessory devices passed through the echoendoscope channel alongside an impacted stent.

Endoscopy_UCTN_Code_TTT_1AS_2AI
